# Causes of the Decay of Teeth

**Published:** 1863-02

**Authors:** 


### CAUSES OF THE DECAY OF THE TEETH.

The frequency with which teeth decay and are lost, and the intimate relation existing between the condition of these organs and the general health, should render a knowledge of the causes of this decay a matter of deep interest to every individual. None are exempt from it, and few indeed seem to escape its ravages.

Many theories were formerly advanced to account for *caries** of the teeth; but only until within the last fifty years have the real causes been understood. To enable the reader better to understand this subject, we will, so far as is necessary for our present purpose, and in as few words as possible, describe the general structure of the teeth, and the agents that operate to produce their decay.

DENTINE. The inner portion of the hard structure of a tooth is called dentine,—tooth-bone—a substance containing more lime, and much harder than the other bones of the body. It is composed of about 72 parts mineral matter (69 of which are lime), to 28 parts animal matter. These proportions vary somewhat in different individuals, and like all the other bony structures, grow harder by an increase of mineral, and corresponding decrease of animal matter, as the individual grows older.

ENAMEL. As a tooth stands in the mouth, the enamel is the only portion of it exposed to view. It serves to cover and protect the *dentine*. It is composed of 99 parts mineral, (94 of which are lime), to 1 part animal matter, and is the hardest of all structures in the animal economy.

With this statement of the composition of the teeth, the reader will more readily appreciate the correctness of the conclusion, when we say that all good dentists, and writers upon this subject, now consider caries of the teeth to be the result of external corrosive agents, acting upon and dissolving, or *eating out*, the earthy portion of their structure.

These agents consist mainly of *acids*, taken into the mouth as medicinal agents, or for pleasure—or originating in a vitiated state of the secretions of the mouth, resulting from an impaired condition of the general health—or from the decomposition of particles of food lodged between and around the teeth.

Such being the cause of decay, the means necessary to be used to prevent it, are apparent. If the *secretions* of the mouth are *vitiated*, this condition should be corrected by proper medical treatment of the general health. The administration of

* Decay.

medicinal agents containing acids, should, if possible, be followed by a cleansing of the teeth, and rinsing of the mouth with a properly prepared alkaline solution. But the great and almost universal cause of the decay of teeth is, *want of cleanliness.* The teeth should be kept clean—*absolutely clean*—as a preventive of their decay and loss, and of large bills with the dentist.

We do not mean to say that attention to these matters will in all cases secure exemption from decay. For, as people must eat and drink, and in seasons of sickness or disability, take substances into their mouths injurious to their teeth, and as some have defective or imperfectly organized teeth, it would be impossible in every case to prevent, entirely, decay at certain vulnerable points. Still, it is true, that a prompt and careful attention to cleansing the teeth after each meal, or as often as anything deleterious is taken into the mouth, would go far, in most cases, to secure freedom from decay, and indemnity against their loss.

We shall endeavor hereafter to point out to our readers the means necessary to be used to keep the teeth clean, and also why it is that some teeth are more liable to decay than others. A.
—*People's Dental Journal.*

